# No difference in endothelial microvasculation measured by peripheral arterial tonometry in patients with Sjögren’s disease and matched controls

**DOI:** 10.3389/fmed.2025.1563796

**Published:** 2025-07-09

**Authors:** Franziska Maria Tapken, Nadine Zehrfeld, Malin Abelmann, Anna Charlotte Müller-Vahl, Sabrina Benz, Tabea Seeliger, Thomas Skripuletz, Torsten Witte, Kristina Sonnenschein, Johann Bauersachs, Udo Bavendiek, Thomas Thum, Anselm A. Derda, Diana Ernst

**Affiliations:** ^1^Department of Rheumatology and Immunology, Hannover Medical School, Hanover, Germany; ^2^Department of Angiology and Cardiology, Hannover Medical School, Hanover, Germany; ^3^Institute of Molecular and Translational Therapeutic Strategies, Hannover Medical School, Hanover, Germany; ^4^Department of Psychology, Ludwig-Maximilians University Munich, Munich, Germany; ^5^Department of Neurology, Hannover Medical School, Hanover, Germany

**Keywords:** endothelial dysfunction, Sjögren’s disease, cardiovascular risk, EndoPAT, peripheral arterial tonometry, miRNA, cardiovascular risk factors

## Abstract

Sjögren’s disease (SjD) is a connective tissue autoimmune disorder characterized by inflammatory infiltration of the exocrine glands, leading to symptoms such as dryness, pain, and fatigue. Additionally, up to 50% of patients may experience extraglandular manifestations. SjD patients face a higher cardiovascular risk, including severe events like myocardial infarction and strokes, partly due to an increased likelihood of subclinical atherosclerosis. Therefore, identifying SjD patients at an early stage is essential to reduce morbidity and mortality. In this study, SjD patients who met the current ACR/EULAR 2016 classification criteria were consecutively enrolled in our outpatient clinic. A control cohort was recruited through a multimedia call for participation. To assess changes in endothelial functions, a reactive hyperemia index (RHI) was calculated using peripheral arterial tonometry with the EndoPAT^®^ measurement device. RHI values below 1.67 were considered pathological. The dataset consists of 49 SjD patients and 27 healthy controls. Both groups had similar ages and comparable cardiovascular risk factors. No differences in RHI were observed between the two cohorts. The only significant factor that was predictive for a low RHI was an increased body mass index (*p* = 0.036). These findings suggest that EndoPAT measurements may not be a suitable method for detecting changes in endothelial function specific to patients with SjD.

## Introduction

Sjögren’s disease (SjD) is an autoimmune connective tissue disorder primarily marked by exocrine gland dysfunction due to lymphocytic inflammation. While it manifests with a variety of symptoms, dryness, pain, and fatigue are among the most common (1). Approximately 30%–50% of patients also exhibit systemic symptoms (2). A recent meta-analysis estimated the prevalence of SjD to be between of 0.01% and 0.05%, identifying no geographical and temporal trends (3).

The underlying pathophysiology of SjD remains incompletely understood. However, chronic inflammation associated with SjD has been identified as an independent risk factor for accelerated atherosclerosis, leading to a notably higher prevalence of cardiovascular disease (CVD) compared to healthy controls (HC) (4–6). The elevated prevalence of CVD-related mortality among SjD patients, combined with an increased incidence of stroke, particularly in patients under 50, underscores the urgent need for early risk assessment to prevent serious long-term complications such as stroke or myocardial infarction (7–9).

Endothelial dysfunction (ED), defined as a deficiency of nitric oxide (NO) that limits the vasodilatory capacity of blood vessels, is one of the earliest detectable stages in the development of atherosclerosis (10, 11).

The present study utilizes the peripheral arterial tonometry EndoPAT^®^ measurement to non-invasively identify microvascular endothelial changes non-invasively in SjD patients compared to HC by inducing reactive hyperemia, which is quantified as the reactive hyperemia index (RHI) (12, 13). EndoPAT^®^ requires minimal training and its accuracy therefore remains almost independent of the examiners’ experience (14). A low RHI value is associated with a higher prevalence of a combined endpoint including cardiovascular death, myocardial infarction, revascularization, or cardiac hospitalizations (15).

Thus, the aim of this study is to detect early microvascular changes in patients with SjD using EndoPAT^®^ measurement. Additionally, the study seeks to evaluate the effectiveness of EndoPAT^®^ as a preventive diagnostic tool.

## Methods

### Study design

This study is a prospective monocentric cohort study that included patients with SjD who routinely visited the rheumatological or neurological outpatient clinic of Hannover Medical School between August 2023 and April 2024. At the same time, a control cohort was recruited through a multimedia call for participation. The study consisted of 76 individuals, with 49 participants diagnosed with SjD and 27 HC, the majority of whom were German and White.

### Participants

All included patients met the 2016 ACR/EULAR classification criteria for SjD. Participants who had been recently pregnant, experienced a malignant cancer within the past 5 years, suffered a myocardial infarction, had a stroke, or were diagnosed with other systemic inflammatory diseases were excluded from the study. All participants provided written informed consent. The study was approved by the local authorities [Institutional Review Board of the Medical University of Hannover approval (8179_BO_S_2018)].

### Data collection

The RHI was measured and automatically calculated using the EndoPAT2000^®^device from Itamar Medical Ltd., Caesarea, Israel. The measurements were performed in the angiological outpatient clinic of the Hannover Medical School. To minimize fluctuations caused by the circadian rhythms, all measurements were conducted at a nearly similar time in the afternoon.

The measurement protocol followed was based on the guidelines established by Axtell et al. (16). Initially, a baseline measurement of 5 min was recorded. This was followed by a 5-min occlusion of the right arm using a manual blood pressure cuff. After the occlusion, the cuff was released, and reactive hyperemia was measured for an additional 5 min. The software from Itamar Medical directly processes these measurements and independently calculates the RHI. According to the company’s recommendations, a RHI value of 1.67 or below was considered pathological. A detailed methodological protocol can be found in the Supplementary Data.

### Statistical and graphical analysis

Data analysis was performed using R version 4.3.1. Descriptive statistics, unless otherwise specified, are reported as median and interquartile range. The SjD patient cohort was compared to the HC group in terms of age, gender, cardiovascular risk factors (CRF), including BMI, arterial hypertension, preexisting hypercholesterolemia, preexisting diabetes, positive family history, HbA1c, serum low density lipoprotein (LDL) and high density lipoprotein (HDL) cholesterol. Continuous variables were analyzed using the Kruskal–Wallis test, while discrete variables were compared using Fisher’s exact test (Table 1).

**TABLE 1:** Baseline demographic data and cardiovascular risk factors in SjD patients and HC.

Characteristics	Control cohort *N* = 27	SjD cohort *N* = 49	*p*-value
Age (years)	62.7 [57.5–65.6]	61,7 [54.2–66.1]	n.s.^a^
Female gender [*n*, %]	22 [81.5]	44 [89.8]	n.s.^b^
Tobacco consumption (pack years)	4.43 [0–30]**	6.10 [0–40]**	n.s.^a^
Body mass index (kg/m^2^)	24.22 [22.3–26.6]	24.97 [22.6–29.4]	n.s.^a^
Arterial hypertension [*n*, %]	6 [22.2]	19 [39.8]	n.s.^b^
Pre-known hypercholesterinemia [*n*, %]	6 [27.3]	16 [32.7]	n.s.^b^
Pre-known diabetes [*n*, %]	1 [0.04]	3 [0.06]	n.s.^b^
Positive family history* [*N*, %]	13 [48.1]	24 [49.0]	n.s.^b^
HbA1c (%)	5.3 [5.1–5.5]	5.4 [5.1–5.6]	n.s.^a^
Serum LDL-cholesterol (mmol/L)	3.48 [2.9–3.8]	3.12 [2.6–3.7]	n.s.^a^
Serum HDL-cholesterol (mmol/L)	1.5 [1.4–2.0]	1.7 [1.4–2.0]	n.s.^a^

Unless otherwise stated median and [inter quartile range] are reported.

^a^Kruskal–Wallis test.

^b^Fisher’s exact test.

*Positive, if first degree relatives were affected by cardiovascular diseases.

**Mean value [range, min–max].

Patients with an RHI value below a threshold of 1.67 were classified as having pathological results according to the manufacturer’s instructions. Furthermore, a Pearson’s Chi-squared test with Yates’ continuity correction was conducted to examine the relationship between two dichotomous variables group (SjD patient vs. HC) and RHI status (pathological vs. not pathological). To compare absolute RHI scores between the SjD- and HC cohort, absolute values were analyzed using a two-sample *t*-test. Fisher’s exact test for count data was employed to assess the association between organ involvement and RHI abnormalities.

The association between RHI and continuous variables was examined using Spearman’s rank correlations. A multiple regression analysis was conducted to determine how well absolute RHI scores can be predicted by disease-related symptoms and CRF. The significance level was set at *p* < 0.05, with all *p* values being two-tailed unless otherwise stated.

Figures were created using R version 4.3.1.

## Results

### Cohort demographics

A total of 49 SjD patients and 27 HC were included in the study population, totaling 76 individuals. The baseline demographic data are summarized in Table 1. There were no statistically significant differences in age or gender between the two groups. Furthermore, there were no relevant differences in CRF such as smoking, diabetes mellitus, and BMI, as shown in Table 2.

**TABLE 2:** Summary of disease related parameters in SjD patients.

Characteristics	*N* [%]
**Disease related parameters**	
Path. Saxon test [43]*	20 [46.5]
Path. Schirmer test [44]*	31 [70.5]
No objective dryness [49]*	12 [24.5]
Chisholm and Mason-grade ≥ 3 [25]*	21[84.0]
ESSPRI-score	5.7 [3.7–7.3]^#^
**Laboratory values at investigation date**	
ANA > 160 [47/49]*	26 [55.3]
Alpha-fodrin antibodies positive [45/49]*	1 [2.2]
Anti-SSA/Ro antibodies positive [47/49]*	30 [63.8]
ANTI-SSB/LA antibodies positive [46/49]*	6 [13.0]
Hypergammaglobulinemia positive [47/49]*	6 [12.8]
Rheumatoid factor positive [47/49]*	11 [23.4]
ESSDAI score [49/49]*	
Constitutional symptoms	4 [8.2]
Lymphadenopathy	2 [4.1]
Glandular involvement	1 [2.0]
Articular involvement	5 [10.2]
Cutaneous involvement	3 [6.1]
Pulmonary involvement	7 [14.3]
Renal involvement	2 [4.1]
Muscular involvement	0 [0.0]
Peripheral nervous system involvement	16 [32.7]
Central nervous system involvement	3 [6.1]
Hematological involvement	11 [22.4]
Biological involvement	10 [20.4]
Total score, points [IQR]	4 [0–10]^#^

Absolute values [relative values] or stated median and [inter quartile range] ^#^ are reported.

*Stated number of available data sets.

Disease activity and organ involvement (OI) were assessed using the EULAR Sjögren-Syndrome Disease Activity Index (ESSDAI) score (17). The median total ESSDAI score was 4 [0–10]. Among patients with OI, the peripheral nervous system was affected in 32.7% of cases. The severity of symptoms, including pain, dryness, and fatigue, was evaluated using the EULAR’s Sjögren-Syndrome Patient-Reported Index (ESSPRI) (18). The median ESSPRI score was 5.67 [3.66–7.33]. Additionally, 63.8% of patients tested positive for SSA/Ro antibodies, while only 13% had a positive result for SSB/La antibodies. The median time since initial diagnosis is 54 months [35–96].

A internal, comprehensive and standardized questionnaire was employed to assess disease-specific symptoms. Alongside the high prevalence of sicca symptoms including dryness of the eyes and mouth (87.8%), the most frequently reported symptoms were arthralgia (75.5%), myalgia (73.5%), and fatigue (69.4%). Furthermore, 38.8% of participants reported experiencing Raynaud’s syndrome, while 28.6% had arthritis. In addition, 22.4% of respondents indicated they had experienced inflammation of the parotid gland, 14.3% reported morning stiffness lasting over 30 min, and 10.2% had suffered from thrombosis in the past.

### Results on RHI

**FIGURE 1:**
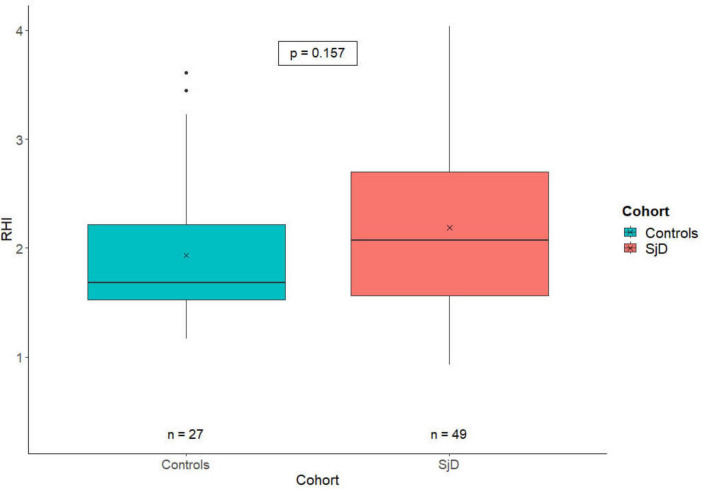
The RHI-value plot depicts the distribution of the RHI in healthy controls (blue) and SjD patients (red). The X indicates the cohort-specific RHI means, with a value of 1.93 for HC and 2.19 for SjD patients. There is only a numerically but no statistically significant difference between the two groups. RHI, reactive hyperemia index; SjD, Sjögren’s disease.

When comparing the RHI scores of HC (*n* = 27) to those of patients with SjD (*n* = 49), there was no significant difference between the two groups (*p* = 0.157). The HC exhibited lower RHI values (*M* = 1.93; SD = 0.67) compared to the SjD cohort (*M* = 2.19; SD = 0.78). The results are illustrated in Figure 1.

Cardiovascular risk factors were assessed for all SjD patients and HC and are summarized in Table 1. Overall, when controlling for all other considered CRF, a significant negative association was found between the body mass index (BMI) and the RHI (β = −0.04; *p* = 0.045), as well as a significant positive association with systolic blood pressure (β = −0.01; *p* = 0.029). The negative association with BMI was also observed when only considering SjD (β = −0.05; *p* = 0.038), after controlling for all other relevant CRF including in this study.

No significant correlation was found between RHI and ESSDAI. RHI showed a tendency toward a negative association with ESSPRI, though this effect was not found to be statistically significant. Similarly, laboratory parameters including SSA/Ro-antibodies, anti-nuclear antibodies and immunoglobulin G showed no significant correlation with the RHI.

## Discussion

Our study is the first to analyze ED using EndoPAT^®^ measurements in a clearly defined cohort of SjD patients within a prospective study design.

**FIGURE 2:**
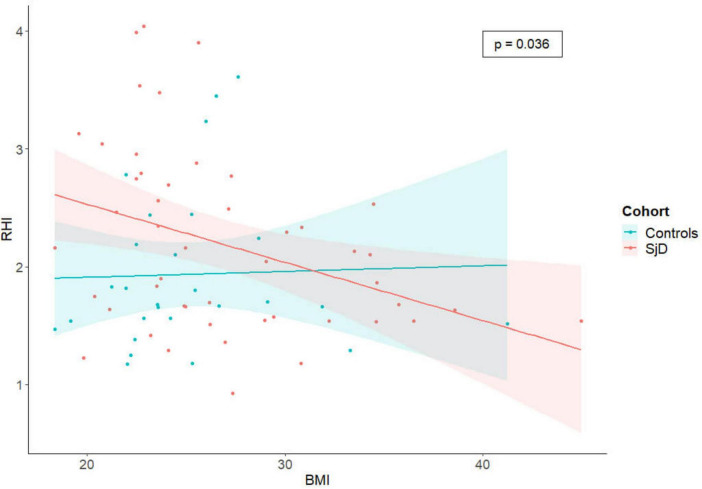
The scatter plot illustrates the correlation between RHI and BMI, with the data subdivided by cohorts. Each individual is represented by a single dot, and a linear regression line has been calculated to illustrate the differences between the cohorts and the significant negative correlation between RHI and BMI in the SjD group, when controlling for all other considered CRF. RHI, reactive hyperemia index; SjD, Sjögren’s disease; BMI, body mass index.

The findings of our analysis indicate that there is no statistically significant difference between SjD and HC cohorts. However after controlling for other relevant CRF, patients with SjD and high BMI exhibited a significantly lower RHI than patients with SjD without obesity (Figure 2). In contrast, this effect was not observed in the HC group. Therefore, it is important to adjust CRF that can be controlled.

In this study, we examined SjD patients without a history of cardiovascular or cerebrovascular events. We wanted to investigate whether ED is already present in the smallest vessels as this could facilitate early identification of patients at increased cardio- and cerebrovascular risk, allowing for prevention or deceleration of atherosclerotic processes.

Sjögren’s disease is associated with an increased risk of cardio- and cerebrovascular events, as well as a higher prevalence of subclinical atherosclerosis (19–21). This association is particularly evident in younger patients, often under 50, who may experience strokes at an earlier age (9). Moreover, patients with SjD exhibit abnormal profiles in novel biomarkers, including microRNAs, which further indicate an elevated cardiovascular risk profile (22).

In light of the fact that atherosclerotic end-stage diseases are a general risk for early death, a risk prevention in patients with an autoimmune disease as an additional risk factor, is highly necessary (23).

Atherosclerosis is a chronic, progressive disease that can remain asymptomatic in its early stages, including cases of ED. ED is characterized by a reduced availability of NO, which impairs the vascular system’s ability to respond with vasodilation (24). This diminished vasodilatory response is reflected in a reduced RHI in the EndoPAT^®^ measurement (16).

A low RHI has shown clinical significance in relation to cardiovascular risk stratification and the early detection of ED (15).

Additionally, ED is linked to an environment that promotes inflammation, cellular proliferation, and increased coagulation, all of which contribute to the development of atherogenesis (10).

Various factors contribute to the development of atherosclerosis. Metabolic syndrome represents one relevant risk factor as a condition that includes obesity among its components, along with impaired carbohydrate metabolism, hypertension and dyslipidemia (25).

Our study has several limitations. Its monocentric design and small sample size limit the generalizability of our findings to the broader population. Additionally, we did not evaluate the impact of general medication use or glucocorticoid intake on vascular tone regulation. By excluding participants with end-stage CVDs, our study may have included patients at a too early stage to detect significant differences between SjD and HC. Consequently, further research is needed to investigate these potential influences.

This research underscores the need for a subsequent longitudinal prospective study to determine whether patients with an abnormal RHI are indeed at an increased risk of cardiovascular events.

## Conclusion

In conclusion, even in the absence of evidence showing early involvement of small vessels or impaired vascular function by EndoPAT, patients with SjD have a known elevated risk for cardiovascular issues. Recognizing this is of particular importance in the context of BMI and the presence of other CRF. Based on our data, we cannot determine whether EndoPAT is unsuitable for detecting differences or if no early differences exist between patients with SjD and HC. Despite the fact that the results were negative, they therefore provide actionable insights that can help to avoid unnecessary tests and focus on more reliable diagnostic procedures such as Doppler sonography or potential biomarkers to detect patients with increased cardio- and cerebrovascular risk at an early stage and treat them preventively.

## Data Availability

The original contributions presented in this study are included in this article/Supplementary material, further inquiries can be directed to the corresponding author.
