# Supplementary material for: No difference in endothelial microvasculation measured by peripheral arterial tonometry in patients with Sjögren’s disease and matched controls
Source: Front Med (Lausanne). 2025 Jul 9;12:1563796. doi: 10.3389/fmed.2025.1563796 (PMC12283599; doi:10.3389/fmed.2025.1563796)

Medizinische Hochschule Hannover

Zentrum Innere Medizin
Klinik für Rheumatologie und Immunologie
Prof. Dr. med. T. Witte, Direktor

Telefon: 0511 532- 6656
Fax: 0511 532- 9067
immunologie@mh-hannover.de
www.mh-hannover.de/kir.html

Carl-Neuberg-Straße 1
30625 Hannover
Telefon: 0511 532-0
www.mh-hannover.de

'Comparison of endothelial function in primary Sjögren's syndrome compared to healthy controls using EndoPat® measurements'

EndoPat® measurements study protocol

Preparations

1. patient preparations in advance
   - Fast for 4 hours beforehand
   - No coffee, tobacco for 8 hours
   - Continue long-term medication as normal
   - Take off watches, rings + jewellery on the fingers

Prior to the procedure, patients were informed of the preparations involved by means of an email communication.

2. materials
   - Armrests
   - Leg roll
   - Cushion
   - Blood pressure monitor
   - Manual blood pressure cuff
   - 2 pneumatic end probes = tubes
   -
3. patient positioning
   - Patients must lie relaxed
   - Arms on the armrests + leg roll under the knees
   - Patients must not speak + move during measurement (risk of artefacts)
   - stop the examination in case of pain

4. querying patient information
   - age
   - height
   - weight

5. patient inspection
   - injuries to the fingers?

6. blood pressure measurement
    - measure RR on the control arm (no tourniquet is applied here later)
    - serves to select the appropriate tourniquet pressure

7. attach manual blood pressure cuff
    - to be attached to the right arm
    - is stowed here during the measurement

8. attach ‘tubes’ to the index fingers of both hands
    - if index finger is not possible, attach to the same side of another finger
    - note orientation between hand and cable
    - sample 1 = right hand
    - sample 2 = left hand

Measurement procedure

1. open the programme
2. patient control ➞ inflate
3. enter patient data ➞ inflate
4. start programme with ‘Go’
4. **Record 5 minutes baseline**: no pressure on manuel blood pressure cuff
5. **5 minutes of occlusion**: approx. 20-30 mmHg (60mmHg) above measured systolic blood pressure, start low, increase until 0 line is visible
6. **Record 5 minutes reactive hyperaemie period** (post occlusion): without pressure
7. end programme + save in study folder + print
8. The software from Itamar Medical directly processes these measurements and independently calculates the RHI.

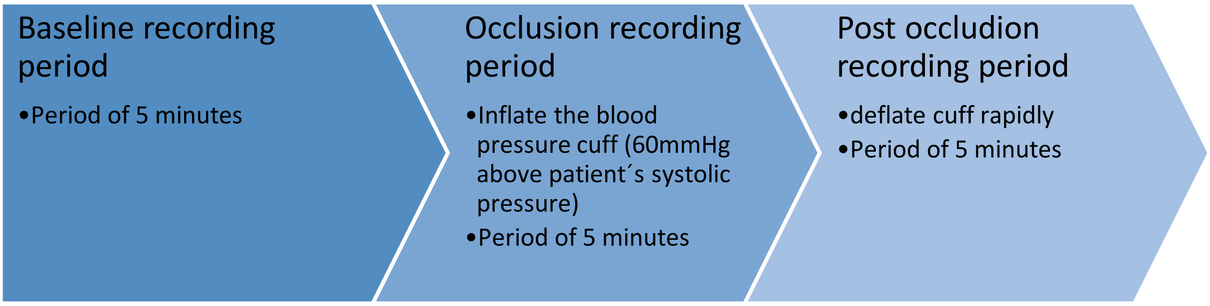

Supplement: Supplementary file 1 [file Data_Sheet_1.PDF]
